# Supplementary material for: Prevalence and risk factors of neurodevelopmental disorders among migrant and refugee preschool children in high-income western countries: A systematic review and meta-analysis protocol
Source: PLoS One. 2025 Nov 24;20(11):e0314333. doi: 10.1371/journal.pone.0314333 (PMC12643264; doi:10.1371/journal.pone.0314333)
Supplement: S1 File — (DOCX) [file pone.0314333.s001.docx]

**Appendix**

**Table 1.** Data extraction template

| **Study Details** | **Population** | **Setting** | **Country of Origin** | **Study Aims** | **Type of NDDs** | **Reported Prevalence** | **Statistical Methods and Risk factors** | **Article’s conclusion/ Recommendations** | **Quality Assessment** | **Author’s conclusion** |
| --- | --- | --- | --- | --- | --- | --- | --- | --- | --- | --- |
| **Authors:**  *Authors et al. [ref]*  **Year of Publication:**  *Example:*2005  **Study design:**  *Example:*  Cross-sectional study  **Country:**  *Example:* Australia  Study period: | **Sampling:**  Details on the number of participants and sampling technique.  **Participants details:**  Details on the characteristics of participants (age, sex, sociodemographic characteristics, etc.).  *Example:* Participants included children of migrants | Details on the setting of the study.  *Example:*  The study was conducted in the City A in Country B, and among participants residing in middle income society. | Details on the country of origin of the participants family.  *Example:*  Participants parent(s)’s country of origin is Country X or Mother is from Country Y. | Details on the aims of the study. | Details on the type of NDDs  *Example:*  Autistic Spectrum Disorder | Details on the reported prevalence.  *Example:*  Prevalence of autism in this population was 8.5%. | Details on the statistical approach used in the study.  Example:  Regression analysis was used to predict factor Z.  **Risk factors**:  Details on the potential risk factor of disease A.  *Example:*  Variable X was a predictor or risk factor of disease X | Details on the Article’s conclusion or Recommendations | **Assessment of methodological quality:** This study has clearly met X/Y (x.x%) criteria in the critical appraisal tool. | Details on the **Author’s conclusions.** |
| **Notes:** Explanation of certain concepts to provide clarity on the interpretation. *Example:* A, B, X and Y are the exemplar used for clarity. | | | | | | | | | | |
