# Supplementary material for: Prevalence and risk factors of neurodevelopmental disorders among migrant and refugee preschool children in high-income western countries: A systematic review and meta-analysis protocol
Source: PLoS One. 2025 Nov 24;20(11):e0314333. doi: 10.1371/journal.pone.0314333 (PMC12643264; doi:10.1371/journal.pone.0314333)
Supplement: Attachment — Submitted filename: Comments to the authors.pdf [file pone.0314333.s003.pdf]

Thank you for inviting me to review this manuscript. This article is well written and the protocol is robust and thorough due to the guidance of the JBI, PRISMA-P, and PRISMA guidelines. The analytical plan is also very robust with important subgroup and sensitivity analyses, and the assessment of publication bias. I have made some comments below for the authors to consider and to hopefully strengthen the manuscript.

Comments/suggestions:

- General:
    - No need to capitalise “neurodevelopment/al” in the middle of sentences
    - It should be “neurodevelopmental disorder”, not “neurodevelopment disorder”
    - There are inconsistencies in how the target population is mentioned (e.g., “<6 years”, “children between 0 and 5 years”, “children under 5 years of age” and “0 to 6 years”). For consistency, clarity, and ease of reading, perhaps stick to one.
- Abstract:
    - Line 52: The second last sentence seems to be incomplete (i.e., “migrant children with and”)
- Introduction:
    - Lines 69-71: The third sentence seems to be incomplete (i.e., “Both genetic and environmental factors contribute to the development of these disorders [2], leading to impairments in motor, cognitive, language, and behavioural [3]”)
    - Lines 100-116: Remove specific research questions or consolidate with Methods section
- Methods:
    - Table 1. Framework for inclusion criteria:
        - I understand that high-income countries were defined as per the World Bank. How have you selected the “Western countries” as this is slightly ambiguous? Were they selected based on geography (i.e., continent, region) and/or cultural, political, or economic characteristics? Do you have a reference?
    - 2.3 Inclusion Criteria:
        - Lines 143-151: Perhaps for succinctness in this section, the description of migrants and refugees should be moved to the Introduction section.
        - Lines 155-157: What about other study designs such as case-control, time-series, or ecological studies?
    - 2.5 Information Sources:
        - Line 184: I believe PsycINFO is also a part of the Ovid platform
    - 2.6 Study Selection:
        - Lines 196-197: Is there any hierarchy for reasons for exclusion for the full-text screening stage?
    - 2.7 Assessment of Methodological Quality:
        - Line 204: It should be “risk of bias”, “calibre” is not a suitable term as it is not informative and non-specific
    - 2.9 Data Synthesis:

    - Before mentioning the weighted inverse variance random-effects model, I would mention that you will perform the meta-analysis if the number of eligible studies and homogeneity permits it.
    - How will heterogeneity be tested?
- Appendix:
  - Table 1: Data extraction template.
    - Maybe be worth including the study period in this table.
