# Supplementary material for: Prevalence and risk factors of neurodevelopmental disorders among migrant and refugee preschool children in high-income western countries: A systematic review and meta-analysis protocol
Source: PLoS One. 2025 Nov 24;20(11):e0314333. doi: 10.1371/journal.pone.0314333 (PMC12643264; doi:10.1371/journal.pone.0314333)
Supplement: Attachment — Submitted filename: Response to Reviewer 2.docx [file pone.0314333.s004.docx]

**Reviewer’s Comment:**

During our assessment of this appeal, we noted that you have decided to restrict your review to manuscripts published in English, without providing scientific justification. There is increasing recognition that exclusion of studies published in other languages can increase the risk of bias in systematic reviews. We feel that any restrictions should be justified based on scientific reasons rather than resource constraints. Therefore, we request that you please address this, and resubmit your manuscript files online once the necessary revisions are complete.

**Response to Reviewer**

First of all, I am grateful to the editorial team for accepting my appeal request and reconsider the manuscript for review.

Thanks for highlighting the issue about restricting the review to manuscript published in English. I have discussed this with other authors and agreed that our review search strategy would not restrict to any language.

I have made necessary changes to the manuscript and uploaded the track changed version for your review.
